# Terpenoids as Potential Anti-Alzheimer’s Disease Therapeutics

**DOI:** 10.3390/molecules17033524

**Published:** 2012-03-19

**Authors:** Ki-Yeol Yoo, So-Young Park

**Affiliations:** 1 Department of Biological Sciences, College of Advanced Science, Dankook University, San#29, Anseo-dong, Dongnam-gu, Cheonan 330-714, Korea; 2 Laboratory of Pharmacognosy, College of Pharmacy, Dankook University, San#29, Anseo-dong, Dongnam-gu, Cheonan 330-714, Korea

**Keywords:** Alzheimer’s disease, therapeutics, terpenoids

## Abstract

Alzheimer’s disease (AD) is one of the most well-known neurodegenerative diseases and explains 50–60% of dementia in patients. The prevalence rate of AD is positively correlated with age and AD affects ≥40% of those over 85 years old. The major AD therapeutics available on the market are acetylcholinesterase inhibitors, such as tacrine and donepezil. New therapeutic agents that can block the disease-inducing mechanisms are essential. Diverse efforts have been made to discover anti-AD agents from natural sources. In this review article, we describe some representative terpenoids such as ginsenosides, gingkolides, and canabinoids as potential anti-AD agents. These compounds exhibit promising *in vitro* and *in vivo* biological activities, but are still waiting clinical trials. Additionally, we also discuss some terpenoids including cornel iridoid glycoside, oleanolic acid, tenuifolin, cryptotanshinone, and ursolic acid, which are under investigation for their *in vitro* and *in vivo* animal studies.

## 1. Introduction

The development of medicine and science has contributed to a dramatic increase in life expectancy worldwide and the average life span may reach 120 years old by 2050. The increasing proportion of the elderly will also be reflected in a marked increase in the number of age-related diseases, including neurodegenerative diseases. 

Alzheimer’s disease (AD) is one of the most well-known neurodegenerative diseases, and explains 50–60% of patients with dementia. The prevalence rate of AD is positively correlated with age, and AD occurs in ≥40% of the elderly over 85 years old [1]. Patients with AD decline in cognitive function and find it difficult to remember recent events during the early stage (short-term memory loss). Once the disease progresses, patients experience difficulties in speech, speaking, and cognitive thinking, which is accompanied by long-term memory loss. Patients suffer from language deficits, depression, aggressive behavior, and psychosis during the late stage and eventually need total care from caregivers.

One of the pathologic hallmarks of AD is senile plaques (SPs), and the major constituent of SPs is beta-amyloid (Aβ), which is surrounded by dystrophic neurites and microglia and accumulates outside of neurons. Aβ is a product of sequential proteolytic cleavage of amyloid precursor protein (APP) by β-secretase and γ-secretase [2]. Aβ accumulates in the brain of patients with AD due to increased production or decreased clearance of Aβ. The overproduction of Aβ found in patients with AD who have genetic mutations in APP (familiar AD) is correlated with early onset (beginning in the 30s) of the disease. An increased amount of Aβ (soluble monomeric form) in the brain self-aggregates into Aβ oligomers (2–6 Aβ peptides) [3,4], which is more toxic to cells than the fibrillar or monomeric form [5]. Therefore, the excess toxic Aβ is the major cause of AD pathology (amyloid hypothesis) [6]. Particularly, the levels of Aβ oligomers correlate with the severity of cognitive impairment in patients with AD and play a critical role in AD pathology [7]. Aggregated Aβ oligomers lead to synaptic dysfunction due to oxidative stress and inflammation [8,9]. A recent study reported that Aβ induces neuronal death by binding to nerve growth factor receptors [10] such as pan neurotrophin receptor (p75NTR) and activation of downstream c-Jun N-terminal kinase signal [11]. In addition, activation of the *N*-methyl-D-aspartate (NMDA)-type glutamate receptor (NMDAR) disrupts calcium homeostasis, eventually inducing oxidative stress and synaptic loss [12,13]. Aβ oligomers bind and modulate presynaptic P/Q-type calcium channels at glutaminergic and gamma-amino butyric acid-ergic synapses and eventually impair P/Q current, which is important for neurotransmission and synaptic plasticity [14,15].

Hyperphosphorylated tau proteins accumulate inside of neurons as a form of paired helical filaments (PHFs), which are known as neurofibrillary tangles (NFTs) [16,17]. NFTs are another pathological hallmark of AD. Tau protein, normally present in neurons, binds to microtubules to promote microtubule assembly and stabilizes microtubules and vesicle transport. Conversely, abnormal hyperphosphorylation to tau significantly reduces the affinity of tau protein to microtubules, so hyperphosphorylated tau aggregates and forms PHFs [18]. Inhibited Wnt signaling following binding to the Frizzled receptor, a Wnt protein acceptor, induces neurotoxic hyper-phosphorylated tau proteins, which are found in NFTs [19]. A report that levels of hyperphosphorylated tau protein in cerebrospinal fluid correlate with the degree of cognitive impairment in patients with AD [20] supports the importance of NFTs in AD pathology. Disease-relevant phosphorylation of tau protein and the aggregation to PHF is induced by Aβ [21,22,23,24]. Additionally, tau phosphorylation is the limiting factor in Aβ-induced cell death [25]. These results support the suggestion that tau phosphorylation plays a critical role in AD progression induced by Aβ.

The major AD therapeutics available on the market are acetylcholinesterase (AChE) inhibitors. A strong correlation exists between the degree of cognitive impairment and a shortage of acetylcholine (ACh) in patients with AD [26]. AChE inhibitors such as tacrine, donepezil, rivastigmine, and galantamine have been developed as pharmacotherapy for AD. Although AChE inhibitors help alleviate AD symptoms, they do not delay disease progression. Therefore, new therapeutic agents that block the disease-inducing mechanisms are essential. Memantine, a NMDA receptor antagonist, has been approved by the U.S. Food and Drug Administration (FDA) to treat AD [27]. This drug improves language function and overall cognitive ability significantly in patients with moderate to severe AD [28,29].

Natural products have been used for medicinal purposes for a long time. The effort to develop natural products as potential therapeutics and advances in extraction and isolation techniques lead to the development of 63% of the natural product-derived drugs from 1981–2006 [30]. Much research effort has been devoted to the development of anti-AD agents from natural sources [31]. Galantamine isolated from bulbs and flowers of snowdrop *Galanthus woronowii* (Amaryllidaceae) has been approved by the FDA as an anti-AD medication due to its inhibitory effect against AChE. Terpenoids such as ginsenosides in *Panax ginseng* (Araliaceae) have been extensively studied to understand their beneficial effects on AD. Among many natural products, the terpenoids are the largest and the most diverse group of naturally occurring organic compounds, which increases the chance that a terpenoid will be identified as having activity against AD. This review article describes some terpenoids as possible therapeutic agents to treat AD.

## 2. Terpenoids

### 2.1. Ginsenosides from *Panax ginseng* CA Meyer (Araliaceae)

**Figure 1:**
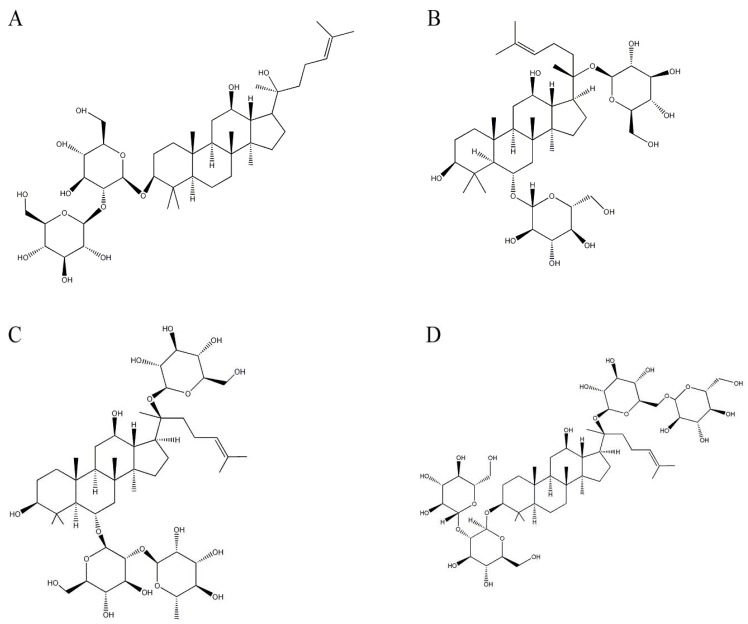
Structures of ginsenosides in *Panax ginseng*. (**A**) Ginsenoside Rg3; (**B**) ginsenoside Rg1; (**C**) ginsenoside Re; and (**D**) ginsenoside Rb1.

Ginsenosides are a series of derivatives of the dammarane-type triterpenes with some sugar moieties attached [32,33], which are the major active components in ginseng isolated from *P. ginseng. P. ginseng* is a well-known traditional medicinal plant that has been used as a representative tonic for thousands of years to promote health and longevity. Among diverse ginsenosides in ginseng extract, ginsenoside Rg3 (Figure 1A) significantly reduces the production of Aβ in CHO2B7 cells by 84% and in Tg2576 transgenic mice by 31% [34]. Ginsenoside Rg3 reduces Aβ levels by promoting Aβ degradation and by enhancing neprilysin gene expression, which is a rate-limiting enzyme in Aβ degradation [35]. Furthermore, ginseng attenuates learning deficits in the damaged or aging brains of rodents [34,36]. Additionally, ginsenoside Rg1 (Figure 1B) attenuates the amount of accumulated Aβ and improves cognitive performance in a transgenic mouse model by activating the protein kinase A/cAMP response element binding protein signaling pathway [37,38]. Ginsenoside Rg1 also reduces Aβ production by modulating the APP process, which is accompanied by an improvement in cognitive function [39,40].

Another ginsenoside in ginseng extract, ginsenoside Re (Figure 1C), protects PC12 cells against Aβ-induced neurotoxicity [39]. In addition, ginsenoside Rb1 (Figure 1D) reverses Aβ-induced memory loss in rats by attenuating neuroinflammation markers in the hippocampus [41]. Ginsenoside Rb1 also exhibits beneficial effects on spatial learning by increasing synaptic density in the brain [42]. 

Ginseng extracts have been studied in clinical trials for their biological efficacy. However, the clinical trial results are complicated by different extraction methods and even different ginseng species used in the different trials. Clinical studies with a placebo-controlled, double-blind, balanced, crossover design have identified both positive and negative effects of ginseng. A 400 mg dose of ginseng extract provides the most beneficial effects in terms of enhanced cognitive function (quality of memory) in a placebo-controlled, double-blind, balanced, crossover design of 20 young healthy adults who are administered a single dose of 200, 400, or 600 mg ginseng (G115) [43,44]. Although these studies have reported significant benefits of ginseng extracts on cognitive function, the small sample sizes limit the certainty of the results. Another clinical trial administers ginseng powder (4.5 g/d) daily for 12 weeks to 58 patients with AD as the treatment group and 39 patients with AD as a placebo control group [45]. Cognitive performance is monitored using the Mini-Mental State Examination (MMSE) score and the Alzheimer’s Disease Assessment Scale (ADAS) during 12 weeks of ginseng treatment. The ginseng group shows gradually improved MMSE and ADAS scores over the 12 weeks of treatment, whereas the control group shows gradually declined MMSE and ADAS scores, suggesting the beneficial effect of ginseng extracts on cognitive function and memory enhancement. However, the beneficial effect of ginseng extract on memory declines gradually to the level of the control group during a 12 week follow-up period without treatment. 

### 2.2. Ginkgolides and Bilobalide from *Gingko biloba* L. (Ginkgoaceae)

**Figure 2:**
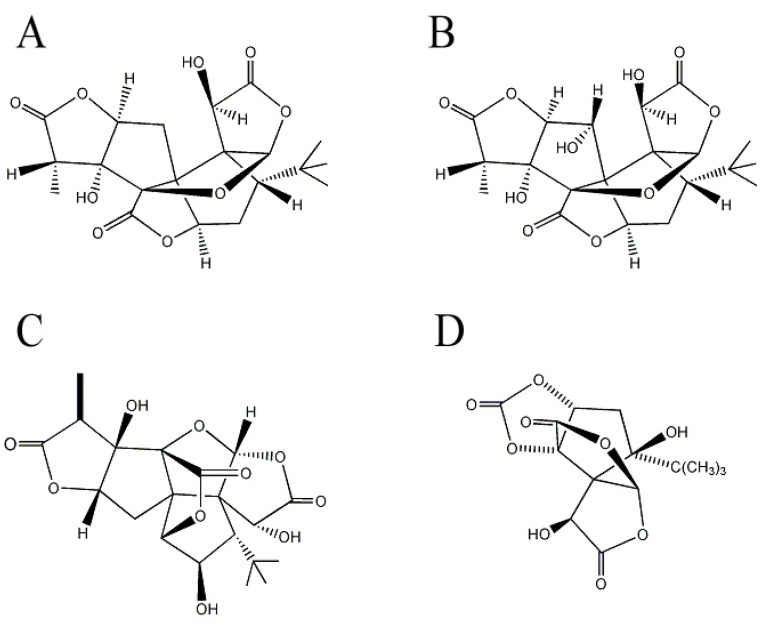
Structures of ginkgolides and bilobalide in *Gingko biloba*. (**A**) Ginkgolide A; (**B**) ginkgolide B; (**C**) ginkgolide J; and (**D**) bilobalide.

Ginkgolides are a cyclic diterpenes of labdane type commonly isolated from *G. biloba*. EGb761, extract of *G. biloba* leaves which contains 24% flavonoid glycosides, 6% terpenoids, and 5–10% organic acids [46] has been extensively evaluated for its neuroprotective effects [47], and terpene trilactones ginkgolides are the major pharmacologically active constituents in EGb761. For example, pre-treatment of neuronal cells with ginkgolide A and B (Figure 2A,B) protects neuronal cells from synaptic damage evaluated by the loss of synaptophysin, a presynaptic synaptic marker [48] and increases neuronal survival against Aβ-induced toxicity [49]. Ginkgolide B rescues hippocampal neurons from Aβ-induced apoptosis by increasing the production of brain-derived neurotrophic factor [50] and reduces apoptotic death of neuronal cells in hemorrhagic rat brain [51]. In transgenic *Caenorhabditis elegans*, ginkgolide A alleviates Aβ-induced adverse behavior including paralysis [52]. Ginkgolide B reverses the Aβ-induced reduction of ACh release from hippocampal brain slices, suggesting potential improvements in learning and memory deteriorated by Aβ [53]. Furthermore, Vitolo *et al*. reports that ginkgolide J (Figure 2C) is the most inhibitor of Aβ-induced hippocampal neuronal cell death among the ginkgolides in EGb761 [54]. Additionally, bilobalide (Figure 2D) reduces Aβ-induced synaptic loss and subsequently enhances hippocampal neurogenesis and synaptogenesis [55]. Bilobalide also rescues chick embryonic neurons from apoptosis induced by serum deprivation or staurosporine treatment [56,57].

Despite these previous findings, regarding the neuroprotective effects of ginkgolides and bilobalides in EGB761 and EGB761 itself, its clinical efficacy is inconsistent and remains controversial. Therefore, clinical trials reflecting diverse races, dementia severity, and different doses of EGb761 should be performed to evaluate the consistency of the effects of EGb761 against AD. 

### 2.3. Cannabinoids from *Cannabis sativa* L. (Cannabaceae)

**Figure 3:**
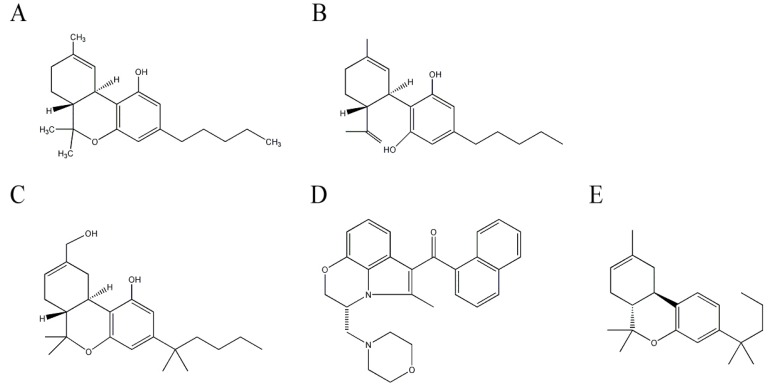
Structures of cannabinoids in *Cannabis sativa* and synthetic cannabinoid derivatives. (**A**) Δ^9^-Tetrahydrocannabinol (THC); (**B**) cannabidiol (CBD); (**C**) HU-210; (**D**) WIN55,212-2; and (**E**) JWH-133.

Cannabinoids are aromatic compounds containing a monoterpene moiety derived from isoprene units, which are isolated from *C. sativa* in the Cannabaceae family. The diverse pharmacological activities of cannabinoids are mediated by activating specific cannabinoid receptors including CB1 and CB2 [58]. CB1 receptors, which mediate the psychoactive properties, are expressed in the nervous system such as neurons and glial cells [59], whereas CB2 receptors are mainly located in immune cells [60]. Δ^9^-Tetrahydrocannabinol (THC) (Figure 3A) mainly binds to CB1 receptors. This plant contains about 60 cannabinoids, and one of the potential anti-AD agents is THC, the major cannabinoid in *C. sativa*. THC is one of the widely-studied natural products and has anti-emetic, anti-convulsive, anti-inflammatory, and analgesic effects [61]. A protective effect of THC against AD has been reported. THC comparatively inhibits AChE and increases the availability of ACh. In addition, THC reduces the inhibition of AChE-induced Aβ aggregation, and subsequently reduces Aβ-induced toxicity [62] and is more efficient than commercially available AChE inhibitors such as tacrine and donepezil. Furthermore, THC reduces behavioral and circadian disturbances in patients with severe dementia [63]. 

Another major cannabinoid having neuroprotective effects against AD is cannabidiol (CBD) (Figure 3B). CBD comprises about 40% of cannabis extracts and is the principle non-psychotrophic constituent. The strong antioxidant effect of CBD provides neuroprotection by reducing oxidative damage such as lipid peroxidation [64,65]. Furthermore, CBD alleviates Aβ-induced inflammatory signals by reducing nitric oxide production by inhibiting p38 and nuclear factor-κB signaling pathways [66]. Tau hyperphosphorylation, one of pathological hallmarks of AD, is also reduced by CBD treatment, as it reduces glycogen synthase kinase-3β, an enzyme responsible for tau hyperphosphorylation in patients with AD [67]. The neuroprotective effects of CBD have been confirmed in an AD-mouse model induced with an intrahippocampal injection of Aβ (1–42) by a reduction in glial activated pro-inflammatory mediators [68,69]. Because CBD lacks psychoactive properties, it is one of the attractive potential anti-AD targets.

Additionally, some synthetic cannabinoids such as HU-210, WIN55, 212-2, and JWH-133 (Figure 3C–E), greatly reduce microglial activation and cytokine production in Aβ-administered rats. Consequently, these synthetic compounds alleviate cognitive impairment by reducing the decrease in neuronal marker levels [69,70,71]. These reports suggest that cannabinoids, particularly THC and CBD, have potential to be developed as anti-AD therapeutics.

### 2.4. Other Terpenoids Having Potential Anti-AD Effects

**Figure 4:**
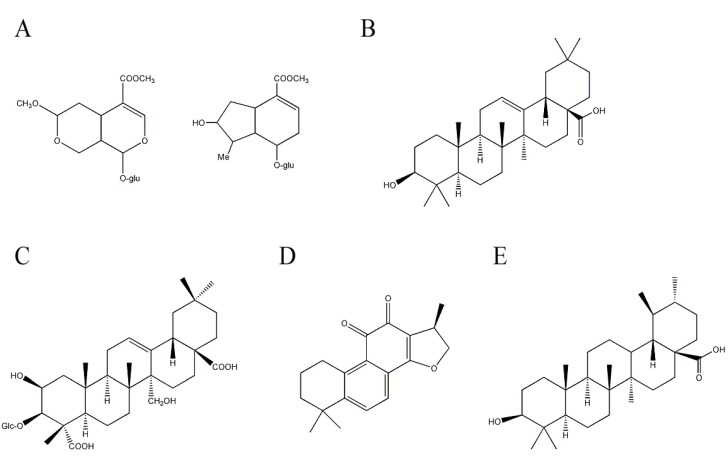
Structures of other terpenoids with anti-Alzheimer’s disease activity. (**A**) morroniside and loganin (**B**) oleanolic acid; (**C**) tenuifolin; (**D**) cryptotanshinone; and (**E**) ursolic acid.

Cornel iridoid glycoside, mainly including morroniside and loganin (Figure 4A) as major compounds in fruits of *Cornus officinalis* Sieb. Et Zucc. (Cornaceae), has a neuroprotective effect by increasing neurological function and decreasing cerebral infarct size in cerebral ischemic rats [72]. Cornel iridoid glycoside improves memory deficits and attenuates hippocampal neuronal loss by improving the brain environment for repair and promoting neuronal survival in fimbria-fornix transfected rats [73].

Oleanolic acid is a triterpene which has been identified as neuroprotective constituents in *Aralia cordata* Thunb. (Araliaceae). *A. cordata* is distributed in eastern Asia such as China, Japan, and Korea and some biological activities including anti-nociceptive, anti-diabetic, anti-oxidant, and anti-inflammatory activities have been reported. Due to its anti-oxidant and anti-inflammatory activities, extracts of *A. cordata* are tested for their neuroprotective effect against Aβ. An extract of *A. cordata* rescues neuronal death induced by Aβ in cultured rat cortical neurons and improves Aβ-induced memory deficit in mice. The neuroprotective constituent included in *A. cordata* is revealed as a triterpene, oleanolic acid (Figure 4B) [74,75].

Tenuifolin is another triterpene that has been reported to be beneficial for AD. It is isolated from *Polygala tenuifolia* Willd. (Polygalaceae). *P. tenuifolia* is a well-known traditional Chinese medicine that is frequently used to improve cognitive function. An extract of *P. tenuifolia* decreases the production of Aβ in *in vitro* cultured cells [76,77]. Additional effort to identify the responsible constituents in *P. tenuifolia* leads to the isolation of tenuifolin (Figure 4C [77]. Tenuifolin reduces Aβ secretion by inhibiting β-secretase, one of the enzymes responsible for cleaving APP to Aβ. Furthermore, tenuifolin improves learning and memory in aged mice by decreasing AChE activity accompanied by increased neurotransmitters levels such as norepinephrine and dopamine [78].

Cryptotanshinone (Figure 4D), a labdane-type diterpene, is an active compound that possesses anti-inflammatory, anti-oxidant, and anti-apoptotic activities [79,80,81]. The compound can be isolated from *Salvia miltiorrhiza* Bunge (Labiatae). Cryptotanshinone easily crosses the blood-brain barrier and affects cognitive function in mice [82]. Furthermore, cryptotanshinone reduces Aβ production by up-regulating α-secretase, which cleaves APPs in the middle of the Aβ sequence, which precludes Aβ production *in vivo* and *in vitro* by activating the PI3K pathway [83,84]. In addition, cryptotanshinone protects neuronal cell damage by inhibiting Aβ aggregation [85]. 

A screening effort to identify potent AChE inhibitors from medicinal herbs leads to the isolation of the triterpene, ursolic acid (Figure 4E) from *Origanum majorana* L. (Lamiaceae). Ursolic acid effectively inhibits AChE activity in a dose-dependent and competitive/non-competitive manner [86]. Ursolic acid also reduces Aβ-induced oxidative damage such as free radical formation and lipid peroxidation in *in vitro* assay systems [87]. Ursolic acid inhibits Aβ binding to microglia, reducing the production of proinflammatory cytokines and neurotoxic reactive oxygen species and leading to a neuroprotective effect against Aβ [88].

## 3. Conclusions

Natural products are attractive sources for developing anti-AD agents, because they can provide diverse structural characteristics and biological activities. Unfortunately, some AChE inhibitors and NMDA receptor antagonists are the only medications approved by the FDA to treat patients with AD. Therefore, this review discussed some natural products and their molecular targets, particularly terpenoids, which can be developed as potential anti-AD agents. The representative terpenoids with anti-AD effects are ginsenosides from *P. ginseng*, ginkgolides and bilobalide from *G. biloba*, and cannabinoids from *C. sativa*. The evaluation of biological activities by *in vitro* cell based assays and *in vivo* animal studies indicate the beneficial effects of these compounds against AD. However, their clinical efficacy is still controversial. Clinical trials should be designed to reflect diverse races, dementia severity, and different doses of biologically active compounds.

Other compounds such as cornel iridoid glycoside, oleanolic acid, tenuifolin, cryptotanshinone and ursolic acid have outstanding neuroprotective effects in *in vitro* assays. These compounds can exert beneficial effects on central nervous system directly or indirectly by acting on peripheral targets. Therefore, the methods to efficiently deliver the bioactive compounds to the brain should be considered to develop terpenoids as anti-AD agents. In addition, the supply of large quantities of biologically active compounds is essential to develop natural product-derived biologically active compounds as therapeutic agents. To overcome this restriction, a mixture of two or three bioactive compounds that act synergistically might be used as the alternative instead of a single compound. Furthermore, a continuous search for bioactive compounds including terpenoids is expected to lead to the discovery of therapeutic agents against AD from natural sources.
